# Postural Tachycardia Syndrome in Children and Adolescents: Pathophysiology and Clinical Management

**DOI:** 10.3389/fped.2020.00474

**Published:** 2020-08-20

**Authors:** Guozhen Chen, Junbao Du, Hongfang Jin, Yaqian Huang

**Affiliations:** ^1^Department of Pediatrics, Peking University First Hospital, Beijing, China; ^2^Department of Pediatrics, The Affiliated Yantai Yuhuangding Hospital of Qingdao University, Yantai, China; ^3^Research Unit of Clinical Diagnosis and Treatment of Pediatric Syncope and Cardiovascular Diseases, Chinese Academy of Medical Sciences, Beijing, China; ^4^Key Laboratory of Molecular Cardiovascular Science, The Ministry of Education, Beijing, China

**Keywords:** postural tachycardia syndrome, pathogenesis, management, children, adolescents

## Abstract

Postural tachycardia syndrome (POTS), characterized by chronic (≥6 months) orthostatic intolerance symptoms with a sustained and excessive heart rate increase while standing without postural hypotension, is common in children and adolescents. Despite the unclear pathogenesis of POTS, the present opinion is that POTS is a heterogeneous and multifactorial disorder that includes altered central blood volume, abnormal autonomic reflexes, “hyperadrenergic” status, damaged skeletal muscle pump activity, abnormal local vascular tension and vasoactive factor release, mast cell activation, iron insufficiency, and autoimmune dysfunction. A number of pediatric POTS patients are affected by more than one of these pathophysiological mechanisms. Therefore, individualized treatment strategies are initiated in the management of POTS, including basal non-pharmacological approaches (e.g., health education, the avoidance of triggers, exercise, or supplementation with water and salt) and special pharmacological therapies (e.g., oral rehydration salts, midodrine hydrochloride, and metoprolol). As such, the recent progress in the pathogenesis, management strategies, and therapeutic response predictors of pediatric POTS are reviewed here.

## Introduction

Postural tachycardia syndrome (POTS) is one of the common forms of chronic (at least 6 months) orthostatic intolerance (OI), and most of the cases have presyncope symptoms accompanied by inappropriate sinus tachycardia with normal blood pressure in an upright position (1, 2). The debilitating condition of POTS is often accompanied by orthostatic discomforts including lightheadedness, headache, giddiness, presyncope, momentary “blackout,” blurred vision, cognitive difficulties, sleep disturbances, fatigue, pale complexion, and even sudden syncope. Postural tachycardia syndrome patients sometimes show signs of excessive sympathoexcitation such as chest tightness, palpitations, inappropriate vasomotor skin changes, excessive sweat, and frequent tremulousness (3, 4). For children and adolescents, the diagnostic criteria of POTS include discomforts of OI together with a normal supine heart rate (HR) and HR increase of at least 40 beats per minute (bpm) or a maximum HR over 130 bpm for children aged 6–12 years or >125 bpm for adolescents aged 13–18 years in the first 10 min of an active standing test or during the passive head-up tilt test (HUTT) without orthostatic hypotension shown by a reduction in systolic blood pressure (SBP) of more than 20 mmHg or a reduction in diastolic blood pressure (DBP) by more than 10 mmHg (5). Postural tachycardia syndrome is a heterogeneous disorder with many possible underlying causes, such as fever, anemia, dehydration, hyperthyroidism, myocardial damage, and autonomic neuropathies. Once a specific cause is identified, the POTS label should be discarded in favor of the appropriate disease term (2).

Because of the multisystem discomforts of pediatric POTS patients, there are significant deleterious effects on individual quality of life (5–8). In pediatric POTS, poor health increases the rate of depression and anxiety in young patients and their parents, which has drawn increasing attention in recent years (9, 10). However, in the general pediatric population, the heterogeneous clinical features and the shortage of specific biomarkers have resulted in an underestimated prevalence of POTS (6, 11). Thus, it is quite difficult to establish the exact incidence of POTS in children and adolescents (6, 11, 12). In 2014, our coworkers enrolled 600 Chinese individuals aged 7–18 (11.9 ± 3.0) years to study the incidence of POTS in children and adolescents, and the results indicated that the prevalence of POTS was 6.8% (4). The results also indicated no significant gender difference among the above young patients (4), although some other studies have reported that most patients with POTS are female (2, 6, 11). Regardless, the data on pediatric POTS are insufficient, meriting multicenter studies to obtain the rate of occurrence of POTS in children and teenagers.

To date, although the precise pathophysiology underlying pediatric POTS remains unclear, there have been some significant reports indicating that POTS might be multifactorial and varied in different subpopulations of young POTS patients (3, 6, 12). Thus, the responses of pediatric POTS patients to the same therapeutic intervention are highly variable (12, 13). Therefore, combined individualized treatment should be established according to the different pathophysiological processes and biomedical predictors (3, 5, 13).

## Physiological Orthostatic Regulation

Under physiological conditions, the blood flow is redistributed when an individual changes position from lying down to standing. Blood volume shifts from the upper part of the body to the lower part and the splanchnic circulation, as well as from the vascular system into the interstitial space owing to gravity while in the upright position (14). Standing reduces venous return, leading to a temporary decline in both cardiac filling and stroke volume (SV) and even a decrease in arterial blood pressure (1, 14).

To compensate for the changes in orthostatism, the body has a series of regulations to restore venous return and cardiac output (14, 15). Importantly, autonomic reflexes are activated. When tension on the vessel walls decreases corresponding to the reduced SV, the baroreceptors of the carotid sinus and the aortic arch can be suppressed to inhibit the parasympathetic response and stimulate the sympathetic response to cause vasoconstriction, accelerated HR, and increased cardiac output (15). The inhibition of baroreceptors also excites the renin–angiotensin–aldosterone system (RAAS) to promote renal reabsorption of water and sodium, which results in arterial vasoconstriction and an increase in plasma volume (16). The compensatory mechanism increases HR by 10–15 bpm, maintains a negligible change in SBP, and elevates DBP by ~10 mmHg (17). Second, muscle pumps are invoked by movement or by external mechanical compression to increase venous return (18). Third, small veins prevent a large accumulation of blood in the lower body and maintain a relative upward flow of blood. Finally, when hypotension is detected, blood pools can be mobilized to the venous system. Additionally, many bioactive small gas molecules and vasoactive peptides are involved in the mechanisms responsible for regulating local vascular tension and endothelial cell function (19). If autonomic compensatory mechanisms are not sufficient, an excessive increase in HR (e.g., POTS) or hypotension, and even a loss of consciousness (e.g. vasovagal syncope) could happen after standing (2, 14).

## Perspectives on the Pathogenesis of Pots

The pathophysiology of POTS is complicated and obscure (3, 12). Current perspectives on the pathogenesis of POTS have been categorized as altered central blood volume, abnormal autonomic reflexes and elevated sympathetic tone, damaged skeletal muscle pump activity, local vascular tension regulation dysfunction, iron insufficiency, mast cell activation (MCA), and autoimmune dysfunction (3, 6, 12).

### Altered Central Blood Volume

Decreased red blood cell volume or low blood volume has been found in many pediatric POTS patients (4, 20, 21). The likelihood of POTS increases 3.9 times if daily water intake is <800 mL, suggesting that hypovolemia is a risk factor in children with POTS (4). In 1996, El-Sayed and Hainsworth (22) confirmed that 24-h urine excretion of sodium was a useful biomarker of the body's volume capacity. In 2014, Zhang et al. (23) showed that there was a negative correlation between 24-h urinary excretion of sodium and symptom severity scores in children and adolescents with POTS. Some investigators have reported that the tachycardic reaction to standing is associated with the seriousness of hypovolemia in pediatric POTS (24) and that symptoms improve following acute (25) or chronic (26) increase in plasma volume. When 24-h urinary sodium excretion is <124 mmol/24 h, salt and water supplementation is efficacious in reducing uncomfortable symptoms in pediatric POTS patients (23). All of the above results indicate that low salt intake compounded by hypovolemia occurs in pediatric POTS. When hypovolemia exists at rest, the body cannot fully compensate for the decrease in blood volume after standing (27), which has to coexist with elevated HR in the upright position (28). Paradoxically, normal plasma volumes in patients with POTS have been reported (29, 30), indicating that hypovolemia is not the unique cause of POTS.

Stewart and Montgomery (31) reported that 37 adolescent patients with POTS, aged 14–21 years, could be classified into a low-flow POTS (LFP) group in 14 patients, a normal-flow POTS (NFP) group in 15 patients, and a high-flow POTS (HFP) group in 8 patients. The blood flow of the lower extremities was measured in the supine position (31). Among these patients, most belonged to the LFP group. The pathogenesis of the NFP group was excessive redistribution to the splanchnic circulation because of inadequate splanchnic vasoconstriction while in an upright posture, leading to thoracic hypovolemia, upright tachycardia, intense peripheral vasoconstriction, and acrocyanosis (32). Interestingly, there were markedly increased microvascular filtration and obviously incompetent peripheral vasoconstriction inducing excessive calf blood flow that was unable to return to the heart accounting for postural tachycardia in the HFP group (33).

In light of the important role of the RAAS in regulating human neurohormones of plasma volume, a perturbed RAAS axis, which is disabled to facilitate the enlargement of blood volume in response to the hypovolemia of POTS patients, might be one of the pathophysiological changes in POTS (34). Several studies have found an inappropriately low plasma renin activity (PRA) in some children and adolescents with POTS, despite the hypovolemia (21, 24, 35), reduced aldosterone levels, and elevated angiotensin (Ang) II levels, with an insensitive receptor response to Ang II (35). Thus, the discordance of Ang II and PRA/aldosterone levels suggested that there might be diminished Ang II degradation in POTS patients (35). Patients with LFP have a decreased body mass index (BMI), which is related to the increased Ang II levels in women aged 14–29 years (36). However, the data on altered RAAS function corresponding to blood volume status in children and adolescents with POTS merit further study.

### Abnormal Autonomic Reflexes and a “Hyperadrenergic” Status

Compared to healthy people, patients with POTS demonstrate excessive blood volume accumulation in the lower extremities and visceral circulation, which results in an obvious decrease in blood volume in venous return to the heart and subsequent cardiac ejection during orthostatic stress (14). The above changes bate baroreceptor activity to excite the sympathetic response and inhibit the parasympathetic response to adjust the altered hemodynamics (3). However, some pediatric POTS patients with a “hyperadrenergic” background have an inability to buffer the decrease in baroreceptor activation, leading to persistent sympathetic excitation characterized by sustained tachycardia while in an upright position (3, 37).

An increase in plasma norepinephrine in the standing position is the main biochemical change that occurs in hyperadrenergic POTS patients (38–40). Zhang et al. (38) reported that the plasma norepinephrine level in the upright position was positively associated with the severity of symptoms and the HR increase in the HUTT in pediatric POTS patients, and they speculated that the orthostatic norepinephrine increase might be due to the decreased vasoconstriction mediated by the baroreflex upon standing. Elevated plasma norepinephrine in pediatric patients with POTS suggests an impaired function of the norepinephrine transporter (NET) (39). Studies have shown that inherited or acquired mutations of NET encoded by the solute carrier family 6 neurotransmitter transporter noradrenalin member 2 (*SLC6A2*) gene impair the removal of norepinephrine in the synaptic cleft and reduce NET-dependent norepinephrine reabsorption (39, 40). The above data suggest that changes in the *SLC6A2* gene or NET protein expression eventually lead to increased adrenaline status in “hyperadrenergic” POTS patients.

Sleeping disorders have been considered to be related to a “hyperadrenergic” status in young patients with POTS (4). Sleepiness, unrefreshing sleep, fatigue, frequent arousal from sleep, and reduced quality of life are often described in pediatric patients with POTS (41–44). However, there is limited evidence to evaluate the prevalence of sleep-related symptoms or to elucidate the mechanism for POTS in children and adolescents (4, 43–45). The probability of POTS in children and adolescents increases 5.9 times if the number of hours of sleep is <8 h/d (4). More than 70% of pediatric POTS patients complain that sleep disturbances are their problems (44). High incidences of sleep-related complaints are not the result of primary sleeping disorders specific to POTS, but a comprehensive effect of complaints such as physical fatigue, chronic body pain, and other somatic discomforts (45). Sleeping disorders often trigger ill-defined cognitive impairment or mental fatigue with increased standing HR, disturbing daytime life in adolescents with POTS (46). Serotonin–norepinephrine reuptake inhibitors can worsen “brain fog” in pediatric POTS patients (47). Thus, exaggerated activation of the sympathicus might be an underlying cause of the hyperaroused state and worsen the subjective estimates of sleep quality in POTS patients (48).

### Damaged Skeletal Muscle Pump Activity

Normally, contraction of the lower extremity muscles commanded by skeletal muscle pumps provides a guarantee for sufficient venous return to the heart to prevent OI (49). Stewart et al. (50) compared 12 LFP subjects with 10 healthy controls and 7 NFP patients to measure venous volume, peripheral blood capacitance, and calf muscle pump function. They found that the reduced circumferences of the calf were associated with a decreased fraction of emptied calf venous volume during muscle contraction concurrent with general low blood flow in LFP subjects. They proposed that the reduced calf blood capacity might lessen calf muscle size in the LFP group and thus damage the vertical ejection ability of the pumps in the skeletal muscle, further reducing blood flow and finally leading to OI in these patients (50).

### Local Vascular Tension Dysfunction and Abnormal Vasoactive Factor Release

Upright stress results in the increased blood filling in the capacitance vessels, followed by reduced venous return (51). Excessive venous pooling has been found in the lower extremities in POTS patients (51, 52), whose resting venous pressures are higher than those of controls (16 vs. 10 mmHg), which causes redistributed blood flow to the lower part of the body even while in a supine position and results in tachycardia via vagal retraction (53). Several studies have attributed such pooling mechanisms to increased venous filling due to arterial inflow (52), increased venous volume owing to mechanical defects in vein (53), blunted arterial vasoconstriction (54–56), altered capillary permeability inducing plasma fluid loss from capillaries to peripheral tissues (33, 57), or impaired venous emptying (58).

To further explore the mechanism for vascular tension dysfunction, some studies have been designed, and the results show that bioactive gaseous regulatory molecules and vasoactive peptides are involved in the development of abnormal partial vascular tension and impaired endothelial cell function in children and adolescents with POTS (5, 35, 59–63). Nitric oxide (NO), an endogenous gas continuously synthesized from l-arginine by nitric oxide synthase (NOS) and constitutively expressed in the endothelium, is known as a vasorelaxant factor and plays a crucial role in regulating the function of vascular endothelial cells (59). Liao et al. (60) reported that there were significantly higher levels of plasma NO and NOS in pediatric POTS patients aged 12 ± 3 years than in age-matched controls. In addition, NOS activity is positively correlated with flow-mediated vasodilation (FMD) of the brachial artery, which indirectly affects vascular dilation function (60). Hydrogen sulfide (H_2_S), a gasotransmitter alongside NO, exerts a low concentration-produced contraction or high concentration-dependent relaxation of blood vessels (61). Erythrocytic H_2_S has been detected to be obviously higher in pediatric POTS patients than that in control group (62, 63). Sulfur dioxide (SO_2_), another gaseous molecule, has recently been found to have vasorelaxant effects (64, 65). Furthermore, plasma SO_2_ levels were significantly higher in children with POTS than in healthy controls and were positively related to the maximum HR of all the study participants (66). The three increased endogenous gaseous molecules in pediatric POTS support the hypothesis that excessive vasorelaxation might contribute to the pathogenesis of pediatric POTS (19, 60). Additionally, adrenomedullin 2/intermedin (AM2/IMD), one of the members of the calcitonin gene-related peptide (CGRP) family (67), has been found to have a positive correlation with extraordinarily high HR during the HUTT in children with POTS (68). C-type natriuretic peptide (CNP) is generated from the endothelium and acts on adjacent vascular smooth muscle cells serving as a selective endothelium-independent vasodilator (69). The potent systemic cardiovascular actions of CNP reduce cardiac filling pressures and heart output following vasorelaxation and decrease venous return to the heart (70). Upright heart output and total peripheral vascular resistance are significantly lower in pediatric POTS patients than in the same children in the supine position or in healthy children and are positively associated with elevated plasma CNP levels (71). The results suggest that the increased endogenous IMD or CNP levels with similar features of vascular dilation represent the endogenous molecules involved in the development of POTS. Serum resistin, known as a new type of peptide hormone derived from adipocytes (72), has been found to notably enhance vasoconstriction and reduce diastolic function (73). An *in vitro* experiment showed that resistin could decrease endothelial NOS expression in human coronary artery endothelial cells (74). The serum resistin level in pediatric POTS patients is dramatically higher than that in age-matched healthy controls and is negatively related to the severity of symptoms and changes in HR that occur when changing from a supine to an upright position (74). These findings suggest that resistin may be a protective element in the pathogenesis of pediatric POTS and that the symptoms of POTS can likely be alleviated by raising the resistin level or improving the resistin function *in vivo* (75). Taken together, these findings confirm that increased local vascular relaxation and the compensatorily increased release of vasorelaxant factors, as well as vascular endothelial cell dysfunction, play important roles in the pathogenesis of POTS in children and adolescents (35, 54).

### Iron Insufficiency

As the richest transition metal ion in humans, iron can cause a decrease in vasodilation by inhibiting the biosynthesis, transportation, transformation, and signal transduction of NO (76, 77). Low ferritin and vitamin D levels have been found in adolescents with POTS (78). Low iron storage accompanied by mild anemia is more prevalent in pediatric POTS patients than in healthy children in the United States (79). Additionally, iron-deficiency anemia also increases NO production in children and adolescents (80), suggesting that low iron storage–induced excessive vascular relaxation by increased output of NO might be a potential pathophysiological factor in pediatric POTS (79, 80). In addition, larger mean corpuscular volume and lower mean corpuscular hemoglobin concentration (MCHC) values were found in pediatric POTS patients than those in controls, which might be associated with decreased iron storage (81).

### Mast Cell Activation

In an evaluation of young females with POTS, Shibao et al. (82) found that some of them had flushing episodes associated with OI, which suggests that MCA is likely involved in the hyperadrenergic mechanism for POTS (82). Although limited studies are available on the detailed mechanisms of MCA in POTS, the histamine, adenosine, platelet-activating factor, and prostaglandins produced by mast cells might play important roles in adolescents with POTS-related vasodilation and tachycardia (83, 84). A hopeful beneficial treatment would be to block these mediators in pediatric POTS patients with MCA, which merits further research (85).

### Autoimmune Dysfunction

Autoimmune disturbance has been considered as one of the mechanisms involved in POTS due to frequent findings of autoantibodies in patients (11, 86, 87). A study from Japan used the luciferase immunoprecipitation system method and detected ~29% anti–ganglionic nicotinic acetylcholine receptor (gAChR) α3 and β4 antibodies from the serum of young POTS population with a median age of 22.2 ± 10.8 years and an onset age of 19.8 ± 10.8 years (88). Dysautonomia may be caused by anti-gAChR antibodies damaging autonomic ganglionic synaptic transmission in POTS patients (89). In addition to gAChR, several adrenergic receptors have been identified in pediatric POTS patients, including Ang II type 1 receptor (AT1R) and β1- and β2-adrenergic receptors (90–92). A study reported that POTS patients had higher autoimmune marker levels [e.g., antinuclear antibody (25 vs. 16%) and antiphospholipid antibody (7 vs. 1%) and increased incidence of comorbid autoimmune disease such as Hashimoto thyroiditis, rheumatoid arthritis, systemic lupus erythematosus, and common variable immunodeficiency (20 vs. 9.4%)] than the healthy population (93). The above findings demonstrate that pediatric POTS has a basis in autoimmune dysfunction; however, the detailed immune mechanisms are unknown and require further evaluation to determine the clinical significance (94).

## Management Strategies for Pots in Children and Adolescents

Full recovery is possible for POTS in children and adolescents, but it is difficult to treat patients because of inconsistent and poor therapeutic efficacies (95). Thus, collaborative and multidisciplinary therapies are constantly required (5–8, 95). After the diagnosis of POTS, in general, health education is the fundamental strategy above all, and further daily treatment plans should consist of non-pharmacological and pharmacological interventions (5, 95, 96). To achieve the best therapeutic effects, the detection of distinct neurohumoral biomarkers might predict the specific efficacy of the individualized treatment options in children and adolescents with specific subtypes of POTS (1, 5, 96).

### Non-pharmacological Interventions

Health education on POTS is needed for young patients and their guardians who should understand the potential precipitating factors and avoid them (5–8, 96). Owing to frequent incapacitating symptoms of POTS that are closely related to an upright posture especially in stifling conditions (2), patients should avoid long periods of standing, sudden head-up postural changes, and high environmental temperatures (5). For POTS patients with a “hyperadrenergic” background, infection, sympathetic activation, and a lack of sleep might aggravate symptoms (4, 38, 39). Thus, patients are prevented from taking drugs such as NET inhibitors or 5-hydroxytryptamine norepinephrine reuptake inhibitors (5). In addition, pediatric POTS patients with sleep disorders should receive a guidance to promote more than 8 h of sleep daily (97). Additionally, it was found that, when the awakening salivary cortisol concentrations were more than 4.1 ng/mL, the sensitivity and specificity of predicting the effect of sleep-promoting therapy were 83.3 and 68.7%, respectively, in patients with POTS (97). Low-flow POTS patients usually suffer from the combination of hypovolemia and muscle pump dysfunction (50), which makes it necessary to drink plenty of water and wear compression garments, which can reduce the venous pooling caused by insufficient peripheral venous reflux and increase peripheral blood return to the heart (98). For those with signs of discomfort, counterpressure actions such as leg crossing and squatting are advised (5).

In addition, appropriate physical exercise to enhance muscle pump function of limbs and autonomic nervous system exercises to improve autonomic tone are also recommended for children with POTS (5, 98). The former refers to regular, organized, progressive exercise plans, which are characterized by aerobic recovery and some resistance training (5). The initial training should be limited to non-upright physical exercises, including the usage of rowing machines, recumbent cycling, and swimming, to reduce standing stress on the heart (5, 98, 99). The latter is especially suitable for pediatric POTS patients whose corrected QT-interval dispersion (QTcd) is more than 43 ms (100). It is recommended that the patients stand against a wall with feet 15 cm from it, starting at 5 min/d, and then gradually increasing to 20 min/d according to patients' tolerance and preference (5, 98). Several training results show that exercise training can improve the symptoms and the quality of life (101–103) and is even better than propranolol in the recovery of upright hemodynamics and the normalization of renal–adrenal reactivity (101).

Children and adolescents with POTS, especially those with 24-h urinary sodium excretion <124 mmol (23) or a BMI <18 kg/m^2^ (104), are encouraged to drink adequate amounts of water (>800 mL/d) (4) and increase appropriate salt intake for 1–3 months to increase blood volume (5). However, this is not advised for patients suffering from kidney disease, high blood pressure, or heart failure (5, 95).

### Pharmacological Treatment

Non-pharmacological interventions should be attempted first in all pediatric POTS patients. If these interventions are ineffective, pharmacological therapies should be applied to solve the specific problems (5, 95, 96). Pediatric patients with POTS who are strongly suspected of having hypovolemia or inadequate salt intake are recommended to use oral rehydration salts (ORSs) for 1–3 months (5, 23, 98, 104) or to accept short-term intravenous saline as rescue treatment for patients with clinical decompensation and significant deterioration of symptoms (25). Saline supplements can alleviate symptoms in “hypovolemic” patients. Many indexes, such as lower MCHC values (81), 24-h urinary sodium excretion <124 mmol (23), and a cutoff of BMI <18 kg/m^2^ (104), have been shown to predict the therapeutic effect of ORSs on children and adolescents with POTS. Additionally, ORSs are also effective when the increase in HR is >41 bpm or when the maximum upright HR in 10 min is >123 bpm before treatment (105). When both indices are used together, the sensitivity and specificity are higher than for any of the single indices (104). Fludrocortisone is useful to increase sodium retention and amplify the plasma volume in POTS patients by activating the RAAS, but its validity needs to be verified in randomized clinical trials (98). In addition, increased NO generation and elevated NOx concentrations can return to normal after oral iron therapy in adolescents with iron-deficiency anemia (80), suggesting that iron supplementation might be effective for POTS patients with iron deficiency and the subsequent vasorelaxation status.

For children with severe symptoms or a risk of injury with unobvious presyncope that remarkably affect the quality of life and for those who do not demonstrate a good response to health training and salt supplementation therapy, special vasoregulation-related pharmacological interventions should be considered (5, 96).

Midodrine hydrochloride, a drug that is enzymatically hydrolyzed as a selective α1-adrenoceptor agonist desglymidodrine by oral administration (106), constricts veins and arteries to increase venous return, which makes the drug theoretically useful in treating POTS (98). Midodrine hydrochloride significantly reduces both supine and upright tachycardia with better results than the α2-adrenoreceptor agonist clonidine, which decreases supine HR without increasing standing HR, but it is inferior to intravenous infusion of saline in POTS patients (25). Midodrine hydrochloride is indicated to be effective for some subtypes of pediatric POTS (5, 107), and its therapeutic efficacy is greatly increased with the help of biomarkers that are low-cost, non-invasive, and easy to detect (108). A series of important studies have been designed to explore the predictors of the efficacy of midodrine hydrochloride in the treatment of pediatric POTS by using receiver operating characteristic curves (62, 109–113). The results show that the following indications can predict the effectiveness of midodrine hydrochloride in the treatment of POTS in children and adolescents: FMD >9.85%, with high sensitivity (71.6% in 1 month and 74.4% in 3 months of therapy) and specificity (77.8% in 1 month and 80% in 3 months of therapy) (109); H_2_S production in erythrocytes >27.1 nmol/min × 10^8^ cell, with a sensitivity of 78.9% and a specificity of 77.8% (62); midregional proadrenomedullin plasma levels >61.5 pg/mL, with a sensitivity of 100% and a specificity of 71.6% (110); copeptin plasma levels >10.5 pmol/L, with both high sensitivity (81.3%) and high specificity (76.5%) (111); and SBP drops in changing from a supine to an upright position, with a sensitivity of 72% and a specificity of 88% (112). Additionally, the blood pressure change from a supine to an upright position can well-predict the long-term asymptomatic survival rate of pediatric POTS patients given oral midodrine hydrochloride (113). Midodrine is contraindicated for those with a BP >95% of the average for individuals of the same age and sex or for those with drug allergies (5). Since midodrine hydrochloride has instant and brief effects, the daily dose is suggested to be divided into several administrations (5, 98, 106). The medicine should be taken only during the daytime, no sooner than 4 h before bedtime, because of a risk of hypertension in the supine position (106); therefore, the BP should be monitored during treatment.

Adrenoceptor blockers can be divided into non-selective blockers for β1 and β2 receptors and β1-selective blockers, which have higher selective affinity for β1 receptors than for β2 receptors (114). The latter mainly slow down HR and decrease myocardial contractility when the sympathetic nervous system is activated but have a relatively small effect on the heart at rest (115). In 2009, a single-center retrospective chart analysis and follow-up conducted by the Mayo Clinic showed that adolescents with POTS taking β-blockers were more likely to have an improvement in symptoms than those taking midodrine (116), suggesting that β-blockers were effective for young POTS patients. β1-Selective blockers are useful for “hyperadrenergic” subtypes of POTS patients (96). There is relatively less experience with β1-blocker therapy in pediatric POTS (5–8, 96). Investigators also proposed that metoprolol, as a common β1-blocker, could be used with an initial dose of 0.5 mg/kg per day in treating pediatric POTS (108) and a maximal dose of 2 mg/kg per day in severe cases according to the symptoms (5). They also found several indicators to predict the effect of metoprolol on POTS in children and adolescents (5, 38, 117, 118). The results showed that the sensitivity and specificity were 95.8 and 70%, respectively, when the plasma CNP level was >32.55 pg/mL (117); 76.9 and 91.7%, respectively, with a plasma norepinephrine level >3.59 pg/mL in upright position (38); and 90.5 and 78.6%, respectively, with a plasma copeptin level <10.2 pmol/L in predicting the efficacy of metoprolol in children with POTS (118). Severe sinus bradycardia, atrioventricular block, severe heart failure, hypotension, acute pulmonary edema, bronchial asthma, mental illness, diabetes, and drug allergy are contraindications for the use of β-blockers (5).

Although evidence hints that MCA and autoimmune disorders take part in the pathogenesis of POTS (82, 86, 88), there are no meaningful therapeutic reports about defending against either of them in pediatric POTS patients, and this research gap requires further study.

## Conclusion

**Figure 1:**
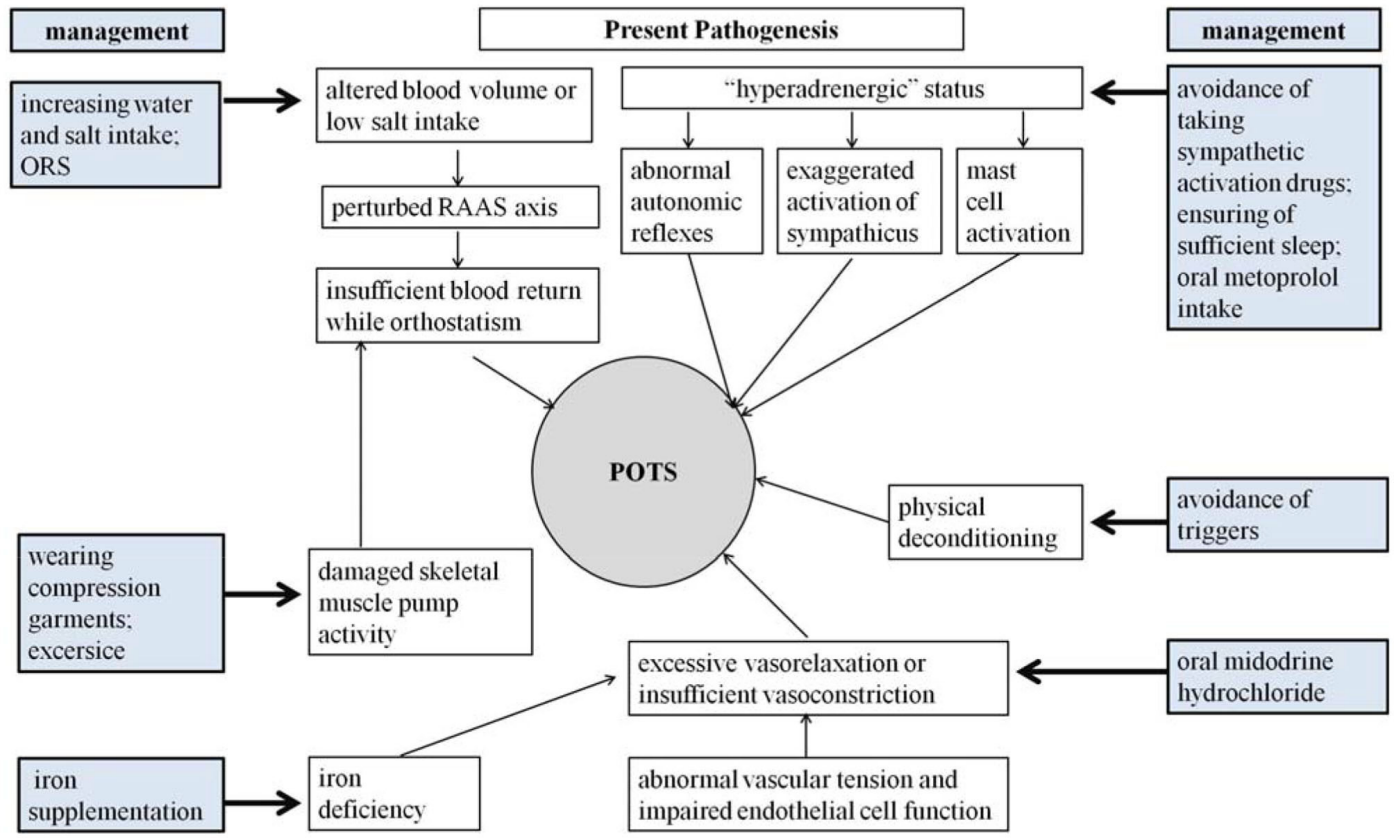
Pathogenesis and corresponding management strategies in pediatric POTS.

Although great efforts have been made in exploring the pathogenesis of pediatric POTS, the precise mechanisms for POTS have not yet been totally elucidated (3, 6, 96). Fortunately, a series of effective treatment measures have been designed according to the known pathophysiological mechanisms (Figure 1), and a number of biomedical factors have gradually been discovered to be involved in the POTS pathological process and treatment prediction (35, 96). More and more biomarkers have been confirmed to be effective in predicting treatment efficacy (5–8). Therefore, selecting individual treatment strategies could improve the therapeutic outcomes according to the detailed pathogenesis indicated by the specific biological indexes (Table 1). However, the data of trials in pediatric POTS patients mainly originate from a single center; therefore, it is essential to perform large-sized multicenter studies in the future to optimize the individualized treatment of POTS in children and adolescents.

**Table 1:** Mechanisms, managements, and therapeutic response predictors of pediatric POTS.

**Mechanisms**	**Managements**	**Therapeutic response predictors**	**References**
Physical deconditioning	Avoidance of triggers	—	(97)
	Sufficient sleep	Awakening salivary cortisol concentrations >4.1 ng/mL	
	Physical exercise	—	
	Autonomic nervous function exercises	QTcd >43 ms	(100)
Hypovolemia	Increase water and salt intake	Lower MCHC values	(81)
	ORS	Urinary sodium <124 mmol/L per 24 h	(23)
		BMI <18 kg/m^2^	(104)
		HR increments of 41 bpm or maximum upright HR of 123 bpm in 10 min	(105)
Damaged skeletal muscle pump activity	Wearing compression garments	—	
Local vascular tension dysfunction	Midodrine hydrochloride	FMD >9.85%	(109)
		Erythrocytic H_2_S production >27.1 nmol/min × 10^8^	(62)
		Plasma MR-ADM >61.5 pg/mL	(110)
		Plasma copeptin >10.5 pmol/L	(111)
		Pre-treatment increase in SBP ≤ 0 mmHg or DBP ≤ 6.5 mmHg from the supine to the upright position	(112)
Hyperadrenergic status	Metoprolol	Plasma CNP >32.55 pg/mL	(117)
		Orthostatic plasma NE >3.59 pg/mL	(38)
		Plasma copeptin <10.2 pmol/L	(118)

*FMD, flow-mediated vasodilation; CNP, C-type natriuretic peptide; MCHC, mean corpuscular hemoglobin concentration; NE, norepinephrine; ORS, oral rehydration salt; MR-ADM, midregional proadrenomedullin; POTS, postural tachycardia syndrome; QTcd, corrected QT-interval dispersion*.

## Author Contributions

GC: literature review, interpretation of data, and first draft of the manuscript. JD: design, organization, and editing of the manuscript. HJ: critical review and editing of studies cited in the manuscript. YH: concept and design of the manuscript, critical review, and editing of the manuscript. All authors: contributed to manuscript revision, read and approved the submitted version.

## Conflict of Interest

The authors declare that the research was conducted in the absence of any commercial or financial relationships that could be construed as a potential conflict of interest.
